# Testing Two Methods that Relate Herbivorous Insects to Host Plants

**DOI:** 10.1673/031.013.9201

**Published:** 2013-09-29

**Authors:** Peter J. T. White

**Affiliations:** 1 McGill University, Department of Biology, 1205 Dr. Penfield Ave., Montreal, Quebec, H3A 1B1, Canada; 2 Current address: Lyman Briggs College, 919 E. Shaw Lane, Michigan State University, East Lansing, MI 48824, USA

**Keywords:** abundance, diversity, host plants, Lepidoptera, species richness

## Abstract

Insect herbivores are integral to terrestrial ecosystems. They provide essential food for higher trophic levels and aid in nutrient cycling. In general, research tends to relate individual insect herbivore species to host plant identity, where a species will show preference for one host over another. In contrast, insect herbivore assemblages are often related to host plant richness where an area with a higher richness of hosts will also have a higher richness of herbivores. In this study, the ability of these two approaches (host plant identity/abundance vs. host plant richness) to describe the diversity, richness, and abundance of an herbivorous Lepidoptera assemblage in temperate forest fragments in southern Canada is tested. Analyses indicated that caterpillar diversity, richness, and abundance were better described by quadrat-scale host plant identity and abundance than by host plant richness. Most host plant-herbivore studies to date have only considered investigating host plant preferences at a species level; the type of assemblage level preference shown in this study has been rarely considered. In addition, host plant replacement simulations indicate that increasing the abundance of preferred host plants could increase Lepidoptera richness and abundance by as much as 30% and 40% respectively in disturbed remnant forest fragments. This differs from traditional thinking that suggests higher levels of insect richness can be best obtained by maximizing plant richness. Host plant species that are highly preferred by the forest-dwelling caterpillar assemblage should be given special management and conservation considerations to maximize biodiversity in forest communities.

## Introduction

Lepidoptera are very important in forest ecosystems. They are an intricate link between forest foliage and higher trophic levels. As larvae (caterpillars) and pupae, they are components of forest food webs, providing an essential food source for birds, small mammals, snakes, amphibians, and other insects. As adult moths, they are food for bats and birds and can be important flower pollinators. Being herbivores in their larval life stage, Lepidoptera play a critical role in forest nutrient cycling, converting nutrient-rich leaves into nutrient-rich feces (either their own or those of a predator) that are easily digestible by soil organisms.

### Overlooked in conservation planning

Even though forest-dwelling Lepidoptera play a central role in forest processes, they are often overlooked in conservation planning (New 2004). One reason they are overlooked is because the natural history of most forest-dwelling Lepidoptera is very poorly known. In temperate regions, research has focused on species capable of outbreak conditions, like the spruce budworm (*Choristoneura occidentalis*), the gypsy moth (*Lymantria dispar*), the forest tent caterpillar (*Malacosome dispar*), or the fall webworm (*Hyphantria cuned*). More cryptic species and virtually all microlepidopteran species have been largely ignored. Acknowledging that individual species-targeted conservation management of Lepidoptera is often not possible. New (2004) suggested that an assemblage level approach could make it easier to make conservation-oriented management decisions leading to the protection of entire lepidopteran assemblages and their natural habitats.

### The relationship between hosts and Lepidoptera

The goal of this study was to determine how host plant richness, abundance, and identity determine Lepidoptera assemblage richness in temperate deciduous forests. Across terrestrial ecosystems, theory has often focused on the richness of host plants driving the richness of Lepidoptera. This builds off the theory that richness at one trophic level determines the richness of the trophic level above (Andow 1991; Rosenzweig 1995). This means that in terrestrial ecosystems, host plant richness would be a logical driver of insect herbivore richness. Indeed, this is what is often reported. In fields, plots with more forb, grass, legume, and woody shrub species have been found to support higher insect herbivore richness and abundance than plots with less plant richness (Siemann et al. 1998; Haddad et al. 2001). A similar pattern has been observed in forest ecosystems, where forest fragments with many tree species sustain higher insect herbivore richness than those with few (Summerville and Crist 2004). Along a successional gradient, insect herbivore richness can be tightly linked to plant species richness in young communities but more tightly linked to structural diversity in old communities (Southwood et al. 1979). The proposed mechanism to explain these types of relationships is that a richer or more diverse host plant community provides more diverse foliar resources and more diverse structural resources than a less diverse host plant community, allowing it to meet the physiological and niche demands of more insect herbivore species (Murdoch 1972; Lawton 1983; Siemann 1998).

The claim that host plant richness drives insect herbivore richness is problematic for two reasons. First, there are many exceptions to this relationship, especially when other factors are tested alongside host plant richness as competing explanatory variables. These factors include site-specific soil nutrient conditions (Hartley and Jones 2003), primary productivity and topography (Hawkins and Porter 2003), and habitat disturbance (Kruess and Tscharntke 2002). In addition, significant differences between insect herbivore richness have been observed among co-occurring host plants. For example, when the black willow tree (*Salix nigra*) and the box elder tree (*Acer negundo*) co-occur, the former tends to host a richer and more abundant Lepidoptera assemblage than the latter (Barbosa et al. 2000).

Similarly, when the Norway maple (*Acer platanoides* L. (Sapindales: Sapindaceae)) and sugar maple (*Acer saccharum* Marshall (Sapindales: Sapindaceae)) co-occur, the former experiences significantly less insect herbivory than the latter (Cincotta et al. 2009). In hybrid zones, hybrid trees can host significantly more (Whitham 1989) or significantly fewer (Boecklen and Spellenberg 1990) insect herbivores than parental tree species, depending on the tree genera and insect assemblage examined. These types of results seem to indicate that higher abundances of certain host plants in forest stands may be more important in facilitating a diverse and abundant insect herbivore assemblage. To my knowledge, this assertion has not been formally tested.

Second, the claim that host plant richness drives insect herbivore richness is actually disconnected from the mechanisms that drive *individual* insect herbivore species abundance and distribution. For individual insect herbivore species, the relationship with host plants is typically described in terms of host plant identity and host plant abundance rather than host plant richness (Thompson and Pellmyr 1991). For example, both the gypsy moth, *Lymantria dispar* (L.) (Lepidoptera: Erebidae), and the winter moth, *Operophtera brumata* L. (Geometridae), are broad generalists, but they tend to have faster developmental rates and higher population abundances when they feed on a select set of preferred host plants (Maufette et al. 1983; Wint 1983; Liebhold et al. 1995; Tikkanen et al. 1999). Many other similar examples exist (e.g., Busching and Turpin 1977; Capinera 1978; Wiklund 1981; Deslile and Hardy 1997; Karban and English-Loeb 1997). These types of preferences are usually driven by host plant-specific foliar nutrient qualities or natural enemy densities, which both have significant impacts on insect herbivore performance and survival (Scriber and Slansky 1981; Thompson and Pellmyr 1991; Hunter and Price 1992). Insect herbivore assemblage richness in a given locale is the culmination of these kinds of host plant choices made by individual species based on the host plants that are present. Since the individual choices are usually made based on host plant identity, insect herbivore richness may be best modeled by taking host plant identity into account.

In this study, two tests were performed to determine how host plants drive insect herbivore assemblage richness in temperate forests. First, whether caterpillar diversity and richness are related to host plant richness was tested. Based on the aforementioned studies (and rationale), an increase in host plant species diversity or richness is expected to be proportional to an increase in insect species diversity or richness. Second, whether caterpillar richness, diversity, and abundance are more accurately described by the abundance of specific host plants was tested. Both positive and negative host plant associations may be expected, synonymous to host plant choices made by individual insect herbivore species and indicative of the presence of preferred and non-preferred hosts. Given that relationships between host plant identity and individual insect herbivore species are often evident, it is reasonable to test whether the cumulative selections made across all species result in detectable preference patterns at the assemblage level. These relationships were tested with a Lepidoptera assemblage in forest fragments of the mixed wood plains in the St. Lawrence River valley of southeastern Canada. This area has historically experienced widespread forest habitat destruction; 85% of the original landscape has been cleared of old-growth forest in favor of agricultural, industrial, and urban development (Allen 2001; Drushka 2003). This type of widespread habitat loss and associated habitat fragmentation can have significant detrimental effects on both generalist and specialist species (Bender et al. 1998). With this in mind, one of the biggest challenges is to manage forest fragments in a way that benefits forest-dwelling species assemblages, maximizing and preserving species richness in intensively developed landscapes.

**Figure 1.**
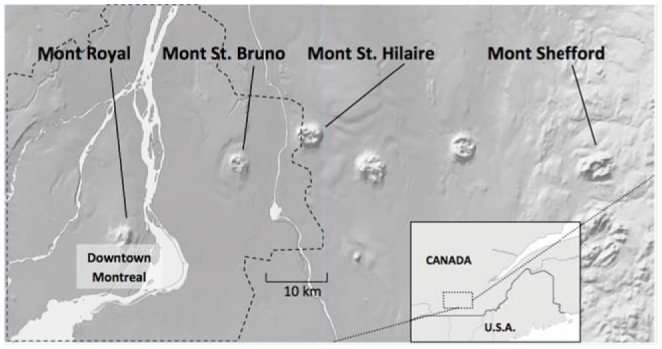
Caterpillars were collected from four sites in the St. Lawrence River valley of southern Quebec, Canada (Figure adapted from Atlas of Canada 2010). The matrix surrounding each site is dominated by agricultural lands and urban development with the exception of Mont Royal, which is a forest fragment in an exclusively urban setting. The dashed boundary represents the Montreal Metropolitan Community. Urban development is very dense in downtown Montreal decreasing towards the Montreal Metropolitan Community boundary. High quality figures are available online.

## Materials and Methods

### Study area

The forest fragments studied were associated with the Monteregian Hills (Feininger and Goodacre 1995) in the St. Lawrence River valley in southeastern Quebec, Canada (45° 30′ N, 73° 30′ W to 45° 24′ N, 72° 35′ W; Figure 1). During pre-settlement times, all the Monteregian Hills would have had broadly similar forests embedded in a more or less continuously forested landscape (Richard and Grondin 2009), but now exist as a series of large forest remnants isolated in the developed landscape. Eighteen 400 m^2^ (20 m × 20 m) quadrats were established in the remnant forests at each of four sampling sites on Monteregian Hills.

The first site was at Pare Mont Royal, an urban park in the middle of Montreal, a city of 3.5 million inhabitants. The forest at the park was cut in the 1950s and early 1960s and was subsequently reforested up until the early 1990s. The land area of the park is 190 ha, ∼100 ha of which is forested.

The second site was at Pare National du Mont St. Bruno, a protected provincial park in the eastern suburbs of greater Montreal. The forest at Mont St. Bruno is a broadleaf deciduous forest covering more than 500 ha of the 790 ha park. Aerial photography records indicate that 60 or more hectares of forest were cut prior to the 1940s in the northern part of the park, and subsequently replanted or allowed to regrow. The forest has more than 85 species of woody plants. Quadrats locations were chosen at random; two were located within the newly reforested area and 16 were located in the older growth areas.

The third site was at the Gault Nature Reserve on Mont St. Hilaire, a protected park and UNESCO Biosphere Reserve 38 km east of Montreal. The forest at Mont St. Hilaire is an old-growth broadleaf deciduous forest covering most of the 1000 ha reserve. This site has a long history of protection dating back to the 1600s (Maycock 1961; Arii 2004).

The fourth site was at Mont Shefford, one of the easternmost Monteregian Hills, 70 km east of Montreal. At the time of this study, different parts of the site were in varying states of disturbance and urban development. Caterpillar sampling was conducted in three sub-sites around the hill. Six quadrats were in a 100 ha semi-disturbed patch of forest on the west side of the Mont Shefford, and six quadrats were in a 25 ha patch of forest on the east side of the hill that is set aside as a forested community park. A quarter of the former site was sugar bush, and there were numerous trails used by deer-hunters throughout the area. The community park had several recreational trails, but off-trail use was discouraged. Six quadrats were in a third sub-site, Parc de la Yamaska, a provincial park just north of Mont Shefford.

### Caterpillar survey and identification

Caterpillars were collected at 18 quadrats at each of the four study sites. Prior to caterpillar surveying, a vegetation analysis indicated that sugar maple, *A. saccharum* Marshall (Sapindales: Sapindaceae), was the most abundant host plant across sites and was the only host plant present at every quadrat. Therefore, at each of the 72 quadrats, 10 sugar maples and up to 10 of all other tree species between 3 and 10 cm diameter at breast height were sampled for caterpillars (Appendix 1). This sampling method was chosen in order to survey a representative proportion of host plants among quadrats. When a given host plant species was abundant in excess of 10 individuals, sample trees were chosen at random. Each sample tree was surveyed by striking the bole and lower branches ten times with a 20 oz, 30” aluminum baseball bat and catching dislodged caterpillars on a 1 m^2^ sheet. Caterpillar collections were made three times at each quadrat (between June 1 and June 6, July 4 and July 9, August 3 and August 6 in 2009). This resulted in a total of 2,090 sampled trees and 62,700 total tree-strikes (2,090 trees × 10 tree-strikes per tree × three caterpillar collection periods).

Macrolepidopteran moth caterpillars were identified to species according to Wagner (2005) with a dissecting microscope. Micro-lepidopteran moths were counted and identified only to morphospecies for lack of an accurate identification guide. Macrolepidopteran moths collected in early instars were reared so that positive identifications could be made.

### Controlling for habitat disturbance

Recent investigations have shown that forest-dwelling caterpillar assemblages are sensitive to intra-habitat disturbances. Any investigation of herbivore-host plant relationships should therefore take this into account. White et al. (2011) showed that there is a consistent negative relationship between recreational trail presence and caterpillar richness in forest fragments in southeastern Quebec, Canada. They suggested that this relationship may be due to increases in caterpillar parasitism/predation and/or changes in trail-side conditions that make trail-side habitat less suitable for caterpillars. Non-native tree species introductions are sometimes correlated to management and can have a negative impact on caterpillar species richness and abundance. To control for these effects, a variable called *Trail Index* was used to measure the impact of trails at each quadrat. *Trail Index* values for the quadrats in this study were described and calculated in White et al. (2011). In short, *Trail Index* is a disturbance coefficient that is geospatially calculated at any given point as the width of an adjacent trail divided by the distance between the point and the trail.

Where multiple trails lie in proximity to a given point total, *Trail Index* is calculated as the sum of the coefficients derived from each trail.

### Analyses

A two-run stepwise (forwards) multiple regression was performed to determine the impacts of trail index and host plant frequencies on caterpillar abundance in the sampled quadrats. This type of analysis is very useful when a large number of independent variables are used and the goal is to eliminate variables of marginal (or non-) significance. While it lacks the sophistication of other multivariate statistical methods (e.g., ordination or regression trees), one advantage it provides is that the final model it computes is independent of insignificant variables. It assumes a Gaussian distribution of the model residual values; collinearity in independent variables can be tested with interaction terms. The decision threshold to include a given independent variable in each step of each regression was based on *p* < 0.05. In the first run-through of the stepwise regression, 25 candidate independent variables were used to explain the variance in (log10 transformed) caterpillar abundances in the 72 study quadrats. This suite of 25 independent variable consisted of the host plant frequencies (24 species, Appendix 2) and the trail-index value of each quadrat. Only host plants that were present at more than two quadrats were included, resulting in the exclusion of 13 of the original 38 host plant species. Although abundant, *A. saccharum* was also excluded from the analysis. First, it is ubiquitous throughout the study region, and the focus was on the impact that additional host plant species had on quadrat caterpillar abundance. Second, *A. saccharum* technically could not be meaningfully included in the regression analyses because they were sampled in equal numbers at each quadrat (i.e., 10 *A*.

After the first run-through of the stepwise regression, a second run-through was conducted using the independent variables selected in the first run-through and the interactions between each of these variables and *Trail Index*. The standard coefficients and partial R^2^ values of the remaining independent variables were then calculated. Two identical analyses were conducted using caterpillar species richness and caterpillar Shannon's diversity (Shannon and Weaver 1949) as the dependent variables in place of caterpillar abundance.

### Host plant and caterpillar relationships

Two simple multiple regressions were used to test significance and strength of the relationship between host plants and caterpillars among quadrats. The first compared caterpillar richness to host plant richness and *Trail Index* (log 10 + 1 transformed). The second compared caterpillar Shannon's diversity to host plant Shannon's diversity and *Trail Index* (log10 + 1 transformed). Interaction terms were included in both models to determine whether there was a relationship between independent variables.

### Testing host plant-specific preferences

The preference for each tree species by the caterpillar assemblage was calculated as a Caterpillar Assemblage Preference Index (CAPI*r*) which measures the observed caterpillar richness in *j* trees of host plant species *i* minus the average caterpillar species richness in *j* trees drawn at random from the entire host plant-caterpillar dataset. It is calculated as:

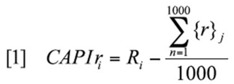
where a given tree species *i* has a cumulative Caterpillar species richness *R* summed across *j* trees that were surveyed. In this calculation, *r* is the caterpillar species richness in a subset of *j* individuals selected at random (with replacement) from the entire dataset of all 2,090 host plant samples from all host plant species; 1000 subsets of *r* were selected and averaged. CAPI*r* is essentially the *actual* caterpillar species richness in host plant species *i* with *j* individuals minus the *average* (i.e., expected) caterpillar species richness found in *j* trees. Thus, if a host plant species with *j* trees has a CAPI*r* value of x, it would be said to support *x* more (or less if *x*, is negative) caterpillar species than would be found if a random sample of *j* trees was sampled from the set of all trees.

**Table 1.**
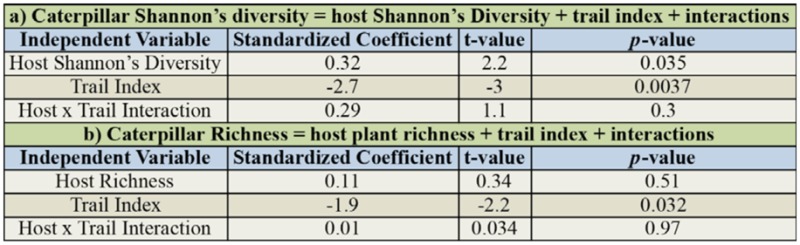
The relationship between host plants and caterpillars shows that (a) host plant (Shannon's) diversity is a significant descriptor of caterpillar (Shannon's) diversity when trail disturbance is accounted for (total model adjusted R^2^ = 0.45 F_3,68_ = 9.9), but (b) host plant richness is a non-significant descriptor of caterpillar richness when trail disturbance is accounted for (total model adjusted R^2^ = 0.27, F_3,68_ = 20.1).

Similarly, the CAPI*r* in terms of caterpillar abundance (CAPI*a*) computes the observed caterpillar abundance in *j* trees of host plant species *i* minus the average caterpillar abundance in *j* trees drawn at random from the entire host plant dataset. It is calculated as:

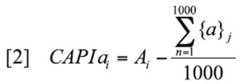
where *A* and *a* are the abundances of host plant species *i* and of a random tree subset withy individuals respectively.

For the purposes of this study, the acceptability of a host plant species is defined as the number of caterpillar species in an assemblage that are documented to use it. Host plant acceptability measures were taken from Wagner (2005) and Handfield (1999). Host plant acceptability was correlated to CAPI*r* and CAPI*a* to test whether caterpillars are distributed relative to the occurrence of acceptable host plants.

## Results

### Caterpillar sampling

1,896 caterpillars were collected, including 53 macrolepidoptera species (1,305 individuals) and 56 microlepidopteran morphospecies (591 individuals) (Appendix 3; Appendix 4 for botanical authorities) from 38 different host plant tree species (2,090 total trees). The 5 most common trees among quadrats (*A. saccharum*, *Fagus grandifolia* Ehrh. (Fagales: Fagaceae), *Fraxinus americana* L. (Lamiales: Oleaceae), *Acer pensylvanicum* L. (Sapindales: Sapindaceae), and *Ostrya virginiana* (Mill.) K. Koch (Fagales: Betulaceae)) yielded 81% of caterpillar catches and high levels of caterpillar richness (Appendix 1).

### Host plant and caterpillar relationships

There was a significant positive relationship between host plant Shannon's diversity and caterpillar Shannon's diversity (Table 1a). *Trail Index* was also a significant descriptor, with the total model explaining 45% of the variance in caterpillar diversity. Host plant richness however was not significantly related to caterpillar richness (Table 1b). *Trail Index* remained a significant descriptor, with the total model explaining 27% of the variance in caterpillar richness.

**Table 2.**
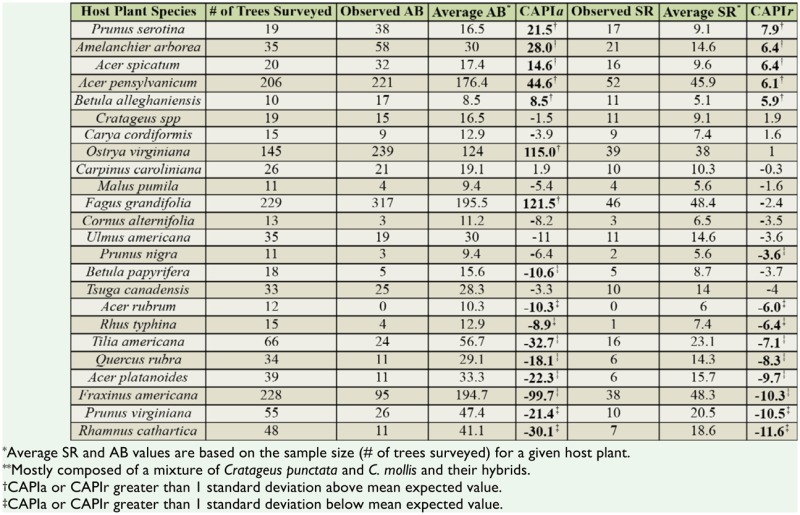
CAPIa and CAPIr values for host plant trees (sorted in order of decreasing CAPIr values) are calculated as the difference between the observed and the average caterpillar abundances and richness in host plant tree species (see equations 1 and 2 in the text). Greater CAPI values indicate that a host plant is more preferred by the caterpillar assemblage.

### CAPIr and CAPIa

**Figure 2.**
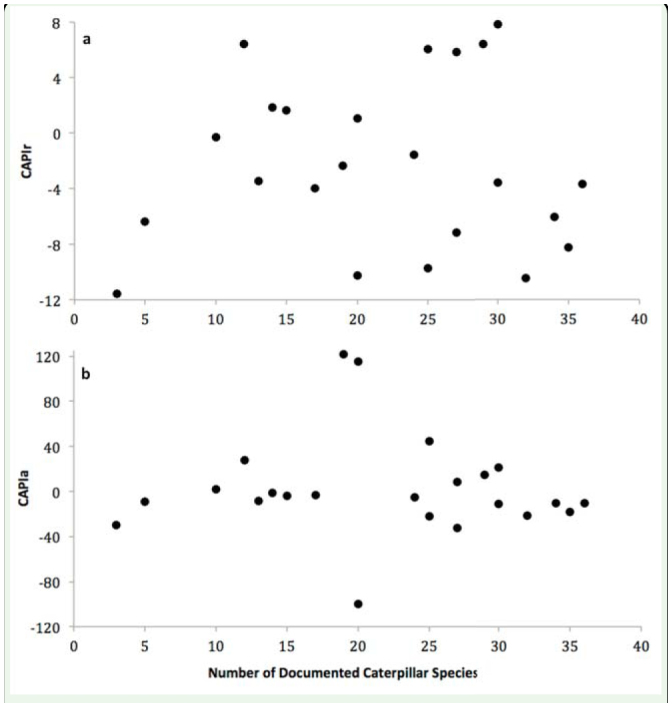
There was no relationship between the number of caterpillars reported to use a given host plant and either (a) CAPIr scores (*p* = 0.91, R = 0.024) or (b) CAPIa scores (*p* = 0.76, R = 0.046). These relationships are expected to be positive, as a host plant's acceptability should be indicative of the caterpillar assemblage preference of that host plant relative to other host plants in the community. High quality figures are available online.

CAPI*r* values ranged between 7.9 (*Prunus serotina* Ehrh. (Rosales: Rosaceae)) and -11.6 (*Rhamnus cathartica* L. (Rosales: Rhamnaceae)); CAPIa values ranged between 121.5 (*F. grandifolia*) and -99.7 (*F. americana*). Increasingly positive CAPI scores indicate that a host plant is used by caterpillars more than average; increasingly negative CAPI scores indicate that a host plant is used by caterpillars less than average (Table 2). There was no detectable connection between acceptable host plants and preferred host plants (Figure 2a, b).

This means that host plant acceptability documented, by Wagner (2005) and Handfield (1999), did a very poor job explaining the variance in CAPI*r* and CAPI*a* (i.e., caterpillar host plant choice).

### Host Plant Importance Relative to Trail Index

For caterpillar abundance, *A. pensylvanicum*, *O. virginiana* and *F. grandifolia* had the highest partial R^2^ values in the step-wise regression. Host plant frequencies accounted for 21.0% of the variance in caterpillar abundance independent of *Trail Index*, which accounted for 19.8% of the variance; this includes a significant interaction between *Trail Index* and *F. americana* frequency (Table 2a). The combination of independent variables accounted for an additional 20.5% of the variance (total R^2^ of model = 0.61).

For caterpillar species richness, host plant frequencies of *A. pensylvanicum* and *O. virginiana* accounted for a combined 24.9% of the variance in caterpillar species richness among quadrats (Table 2b). *Trail Index* accounted for an additional 22.3% of the variance; this includes a significant interaction between *Trail Index* and *F. americana* frequency. The combination of independent variables accounted for an additional 11.5% of the variance (total R^2^ of model = 0.59).

For caterpillars Shannon's diversity, host plant frequencies of *A. pensylvanicum*, *O. virginiana*, and *Ulmus americana* L. (Urticales: Ulmaceae) accounted for 27.9% of the variance in caterpillar diversity (Table 2c). Trail Index accounted for an additional 18.7% of the variance (this again includes a significant interaction between *Trail Index* and *F. americana* frequency); the combination of independent variables accounted for an additional 9.4% of the variance (total R^2^ of model = 0.56).

## Discussion

Host plant identity and abundance were statistically significant and strong predictors of caterpillar richness. This was contrasted by host plant diversity, which had a statistically significant, but very weak, effect on caterpillar diversity. Tree richness and caterpillar richness were unrelated. These results are in sharp contrast with the idea that host plant richness drives insect herbivore richness (Southwood et al. 1979; Lawton 1983). Instead, the results indicate that host plant identity and abundance are more appropriate measures for explaining insect herbivore assemblage diversity, richness, and abundance. This is a novel result that has not yet been described in analyses examining the relationship between host plants and insect herbivores.

### Biodiversity and conservation

The results suggest that reserve management should adopt an approach that identifies and promotes high biodiversity host plants. This is in contrast with other popular approaches, such as maximizing stand structural complexity, maximizing floral biodiversity, and using natural disturbance regimes (Battles et al. 2001; Lindenmayer et al. 2006). Niemela and Neuvonen (1981; Neuvonen and Nimela 1983) suggested that the most important host plants in temperate forests for Lepidoptera biodiversity are those with the highest abundance. This is true in a static sense; in most northeastern broadleaved forests, the *A. saccharum* is the most important for insect herbivores. By virtue of being the most abundant tree, it hosts the highest insect herbivore species richness. But, this is a narrow view that does not take into account low abundance host plants. In situations where reserve management has a mandate to manage tree relative abundances to maximize overall forest health and biodiversity, a more nuanced approach is warranted. For the range of host plants in northeastern deciduous forest, this suggests that restoration and replanting efforts should include black cherry (*P. serotina*), serviceberry (*Amelanchier spp*.), mountain maple (*Acer spicatum*), striped maple (*A. pensylvanicum*) and yellow birch (*Betula alleghaniensis*). In regions where different forest types persist, a system-specific analysis of tree hosting abilities should be conducted to identify high and low biodiversity host plants. Pair-wise host plant comparisons can be useful for this purpose and have been conducted for many common plants (Barbosa et al. 2000; Cincotta et al. 2009). That said, there is evidence to suggest that herbivore-hosting capabilities of trees can be conserved across large geographic scales. Moran and Southwood (1982) found that the relative species richness of insect herbivores and insect predators were very similar on five tree taxa present in both the United Kingdom and South Africa. This might suggest that the preference indices calculated for broadleaved deciduous forests in southern Canada may be broadly applicable to maple-dominated broadleaved and mixed-wood forests across northeastern North America.

### Low caterpillar richness and abundance in invasive trees

The results also indicate that invasive trees may be problematic in deciduous broadleaved forests. The impoverished caterpillar assemblages found on *A. platanoides* (Norway maple) and *R. cathartica* (European Buckthorn) add to a growing body of evidence showing that non-native Eurasian host plants are a detriment to native North American forest insect assemblages (Niemela and Mattson 1996). *A. platanoides* was introduced in the late 1700s (Spongberg 1990) and has periodically been planted for forest restoration (Webb and Kaunzinger 1993; Larson 1996). However, it often outcompetes native tree species and is able to invade intact woodlands (Bertin et al. 2005; Wyckoff and Webb 1996). The results of my study reinforce the trend identified by Cincotta et al. (2009) showing that *A. platanoides* is not a favored host plant of forest insect herbivores. Their results compared *A. platanoides* to *A. saccharum*, whereas I showed it in relation to other common sub-dominant host plant trees, where it ranked 21^st^ among 24 tree species for both CAPI*r* and CAPI*a* (Table 3). Similarly, *R. cathartica* ranked 24^th^ and 22^nd^ in CAPI*r* and CAPI*a*, respectively. Research has shown that *R. cathartica* is a detriment to forest communities as it modifies soil nitrogen conditions, reduces leaf litter levels, propagates the spread of invasive species, is not consumed by many native herbivores, and has allelopathic effects on native trees (Heneghan et al. 2004; Knight et al. 2007). While *R. cathartica* can be beneficial for sustaining insect populations in disturbed and urban settings (VanVeldhuizen et al. 2005), its negative association with forest-dwelling moth populations give further reason for its control in North American forest fragments (Gassmann 2005; Moriarty 2005). Curiously, *F. americana* also had markedly low CAPI*r* and CAPI*a* scores, ranking 22^nd^ and 24^th^ (respectively) among 24 tree species. Species in the genus *Fraxinus* tend to be higher than average in terms of leaf toughness (Ricklefs and Matthew 1982), support high caterpillar parasitoid loads (Lill et al. 2002), and can have prohibitively toxic phenolic compounds (i.e., in the case of the closely related *Fraxinus pennsylvanica*; Markovic et al. 1996). Despite these, they are, however, documented as widely-used host plants by caterpillar species (Ricklefs 1984; Handfield 1999; Karban and Wagner 2005). The stepwise regression analysis indicated that *F. americana* has a strong association with trails at the study sites, which, when combined with these other deterrents, may have resulted in the low CAPI scores.

**Table 3.**
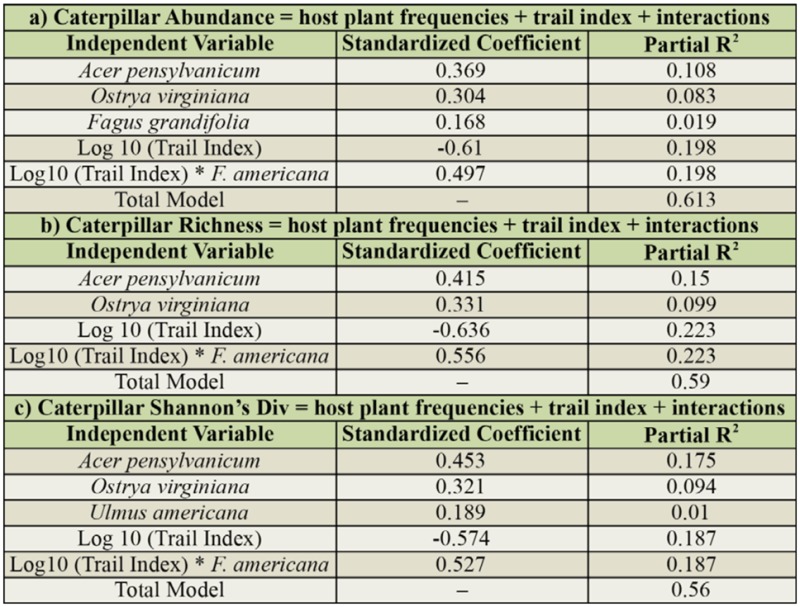
Host plant frequencies (combined) explained (a) 21.0% of caterpillar abundance, (b) 24.9% of caterpillar richness, and (c) 27.9% of caterpillar Shannon's diversity among quadrats. This was independent of *trail index* which, when combined with a *Fraxinus americana* interaction term, explained (a) 19.8%, (b) 22.3% and (c) 18.7% of the variances.

**Figure 3.**
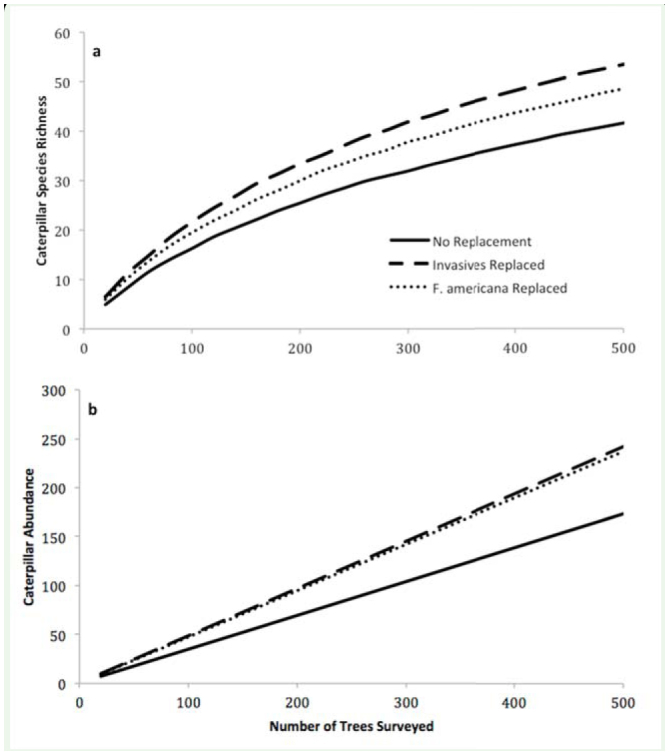
A host plant replacement simulation tor (a) caterpillar species richness and (b) caterpillar richness in the Mont Royal forest fragment. In these simulations *Fagus grandifolia* and *Acer pensylvanicum* were substituted for *Acer platanoides* and *Rhamnus cathartica* (dashed line), and *Ostrya virginiana* was substituted for *Fraxinus americana* (dotted line). The substituted species were chosen because they had high CAPIr and/or CAPIa scores and commonly share the same general canopy position as the species they replace. Replacement of invasive species with *O. virginiana* and *A. pensylvanicum* resulted in an increase of 30% in caterpillar species richness and 40% in caterpillar abundance. Replacement of *F. americana* with *F. grandifolia* resulted in an increase of 18% in caterpillar species richness and 37% in caterpillar abundance. High quality figures are available online.

From the point of view of forest management for biodiversity conservation, the full impact of *A. platanoides* and *R. cathartica* on forest dwelling moth assemblages can be enumerated with a host plant replacement simulation. At Mont Royal, caterpillar surveys included a total of 629 sampled host plants, the most abundant being *A. saccharum* (180 trees surveyed), *F. americana* (102 trees surveyed), *R. cathartica* (48 trees surveyed), *T. americana* (48 trees surveyed), and *A. platanoides* (39trees surveyed). The remaining 212 surveyed trees were made up of 26 different host plant species. Caterpillar species richness and abundance collector curves can be created (using the second half of equations 1 and 2, respectively), first using all trees at Mont Royal and second by replacing the data collected from *A. platanoides* and *R. cathartica* on Mont Royal with data collected from *O. virginiana* and *A. pensylvanicum* from other locations (Figure 3). This results in an increase of 30% in caterpillar species richness and 40% in caterpillar abundance. In this exercise, *O. virginiana* was chosen as a replacement for *A. platanoides* because it had a similar abundance (averaged per site) and average height (5.6 meters versus 5.5 meters). *A. pensylvanicum* was chosen as a replacement for *R. cathartica* because these species are both often subdominant trees associated with disturbed areas (sun-loving) and are relatively similar in average height (5.4 meters versus 3.8 meters). A similar replacement simulation can be run for *F. americana*. While native, its CAPI*r* and CAPI*a* scores were among the lowest in the host plant data set. When the caterpillar data collected from 102 *F. americana* surveyed at Mont Royal were replaced with data from *F. grandifolia* (both are common canopy-contributing species and have similar average heights, 7.4 meters versus 6.9 meters) caterpillar species richness increased 18%, and abundance increased 37% (Figure 3).

### Mechanisms

Given the important implications of these results, it is worthwhile to consider the ecological mechanisms that may be driving the observed relationships. Since host plant choices are made by individual Lepidoptera species (and scale up to the assemblage level), any driving mechanism explaining the assemblage-wide pattern would need to beoperational at the individual species level. The most well established factors used to explain host plant choices among individual species are host plant foliar quality (Feeny 1970; Mattson 1980; Buse et al. 1998) and the presence of natural enemies (Hunter and Price 1992; Siemann et al. 1998; Lill 2001).

Nitrogen has been identified as one of the preeminent foliar nutrients associated with insect herbivore host plant selection and performance (Mattson 1980). This makes it a prime foliar candidate to explain the host plant preferences observed in the present study. One of the most preferred host plant species in this study, *O. virginiana*, is documented as having moderate to high levels of foliar nitrogen compared to other broad-leaved species (Mertzger 1990). However, the widely preferred host plant *A. pensylvanicum* has a moderate to low level of foliar nitrogen (Zehnder et al. 2009) similar to that of the avoided host plant *F. americana* (Abrams and Mostoller 1995). One of the most nitrogenrich host plant species, *Betula papyrifera* Marshall (Fagales: Betulaceae) (Abrams 1998), also had lower than average CAPI*r* and CAPI*a* values (Table 3). This finding would be consistent with the findings of Karban and Ricklefs (1984), who found no relationship between foliar nutritional quality and caterpillar species richness and abundance in broadleaved deciduous caterpillar communities. Although a simple relationship between CAPI values and foliar nitrogen content does not seem apparent in this case, other foliar variables such as water content (Scriber and Slansky 1981) and foliar toxins (Gatehouse 2002) have been commonly cited as determinants of insect herbivore performance and distribution. In fact, the interaction between insect herbivores and foliar toxins across evolutionary timescales has been identified as a potential driver of modern host plant preferences(Scriber 2002; 2010). If a caterpillar species evolves the ability to overcome the toxins in a particular host species, it may also then be able to overcome the chemical defenses of closely related host plant species (e.g., within the same genus). This can result in the adaptive radiation of a group of closely related oligotrophic caterpillar species that utilize a group of closely related host plant species (Ehrlich and Raven 1964; Fordyce 2010). Coevolution of specialist species and their host plants has also been documented (Edmunds and Alstad 1978; Agrawal 2005). Although the majority of caterpillars in the study system were broad generalists, these types of historical relationships between herbivores and hosts may explain why certain caterpillar species preferred certain hosts. In an in-depth analysis of host plant-insect interactions, Futuyma and Gould (1979) concluded that the variation in insect populations among hosts is likely due to a multiplicity of plant leaf chemical variables.

Top-down pressure from parasitoids can also have a significant impact on oviposition host choices of adult Lepidoptera (Thompson and Pellmyr 1991; Karban and English-Loeb 1997). Lill et al. (2002) documented parasitoid-host plant-caterpillar interactions in common host plant genera and showed that the genera *Fraxinus* and *Acer* were associated with higher than expected caterpillar parasitoid loads, while the genus *Ulmus* was associated with lower than expected caterpillar parasitoid loads. They did not include *Ostrya* or *Fagus* in their analyses. The positive association between *Fraxinus* and caterpillar parasitoids could explain the exceptionally low *F. americana* CAPI*r* and CAPI*a* values. However, *U. americana* also had lower-than average CAPI*r* and CAPI*a* values, even though this genus is a documented predator-reduced space. This discrepancy could indicate that parasitism was not the primary driving mechanism of caterpillar assemblage richness in *Ulmus* hosts in the study region. The wide range in CAPI*r* and CAPI*a* values for different *Acer* species in the data further suggest that a genus-level parasitoid-control of caterpillar assemblages may not be the dominant driver impacting caterpillar hostplant preferences. For example, *A. pensylvanicum* and *A. spicatum* had significantly positive CAPI*r* values (6.1 and 6.4 respectively), but *A. rubrum* and *A. platanoides* had significantly negative CAPI*r* values (-6.1 and -9.7 respectively). Similarly, *A. pensylvanicum* and *A. spicatum* had significantly positive CAPI*a* values (44.5 and 14.6), but *A. rubrum* and *A. platanoides* had significantly negative CAPI*a* values (-10.3 and -22.3 respectively). If parasitoid regulation of caterpillars was occurring at the host plant genus level, then species within a given genera would be expected to have roughly similar CAPI*r* and CAPI*a* values. While it is still possible that host plant caterpillar parasitoid loads may play a role in driving caterpillar assemblage richness among the other host plant genera, it seems implausible that they were the sole driving mechanism determining caterpillar species richness and abundance among *Acer* or *Ulmus* host plants in the study.

### Conclusion

Understanding the important determinants of insect assemblage richness and abundance in remnant forest fragments can improve management and conservation efforts. In a landscape where pristine forest habitat is rare, conservation-based management should attempt to maximize and maintain the richness in the forest fragments that remain. In this study, it was shown that richness and abundance of an insect herbivore assemblage can be more effectively described in terms of host plant identity and host plant abundance. Describing the insect herbivore assemblage in these terms is more consistent with single-species studies that show insect herbivore host choice is often a function of host plant identity rather than quadrat- or stand-scale host plant richness. In the system I examined, it did not appear as though top-down parasitoid control was the driving force behind host choice at the herbivore assemblage level (Lill et al. 2002). It also seemed unlikely that host plant foliar nitrogen content was driving assemblage level host choice. Given that neither of these two mechanisms seemed dominant, it is possible that the assemblage-level host plant selections were driven by a complex interaction of multiple foliar nutrient properties and top-down pressure (Mayhew 1997). It is also possible that there was a third factor (e.g., historical disturbance events) driving both the relative abundance of community host plant species and Lepidoptera assemblage host plant occupancy. In a direct conservation application of the results, host plant replacement simulations indicated that planting preferred host plants in the place of non-preferred host plants could result in a profound impact on insect herbivore assemblage richness and abundance. At a broader level, these results call for a shift in conservation management principles where some emphasis should be placed on identifying and protecting high value host plants that are synonymous with high levels of insect herbivore richness and abundance.

## Figures and Tables

**Appendix 1.**
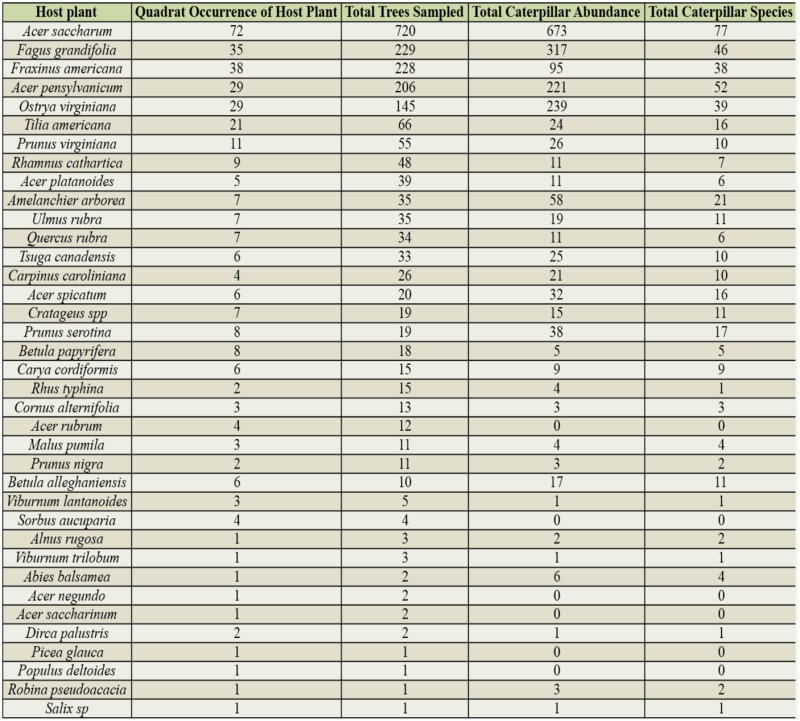
Caterpillar collections from 38 host plant trees in 72 quadrats across four study sites.

**Appendix 2.**
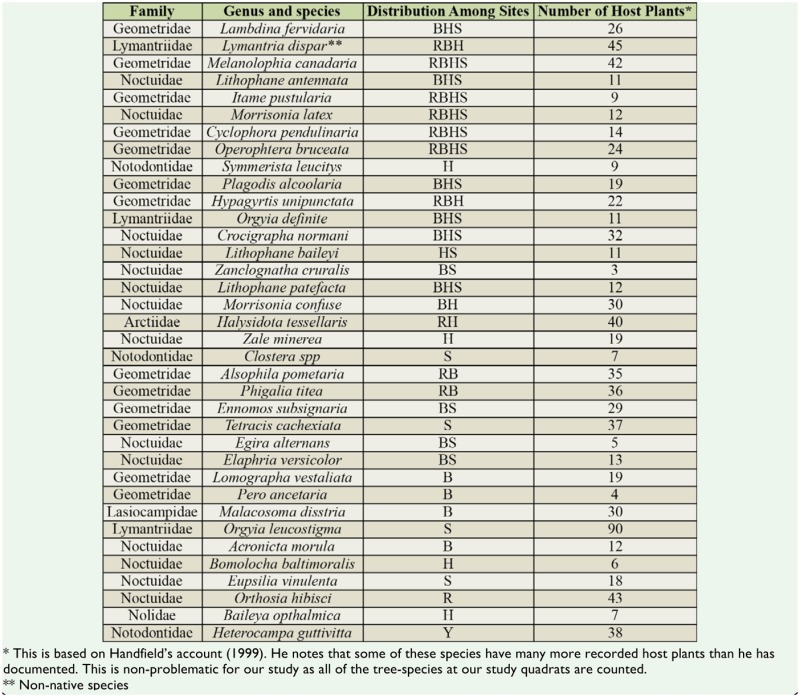
Macrolepidopteran moth species were surveyed across the four sites in the study regions of Mount Royal (R), Mont St. Bruno (B), Mont St. Hilaire (H), and Mont Shefford (S). All species IDs were based on fifth or sixth instar larvae identified using Wagner (2005).

**Appendix 3.**
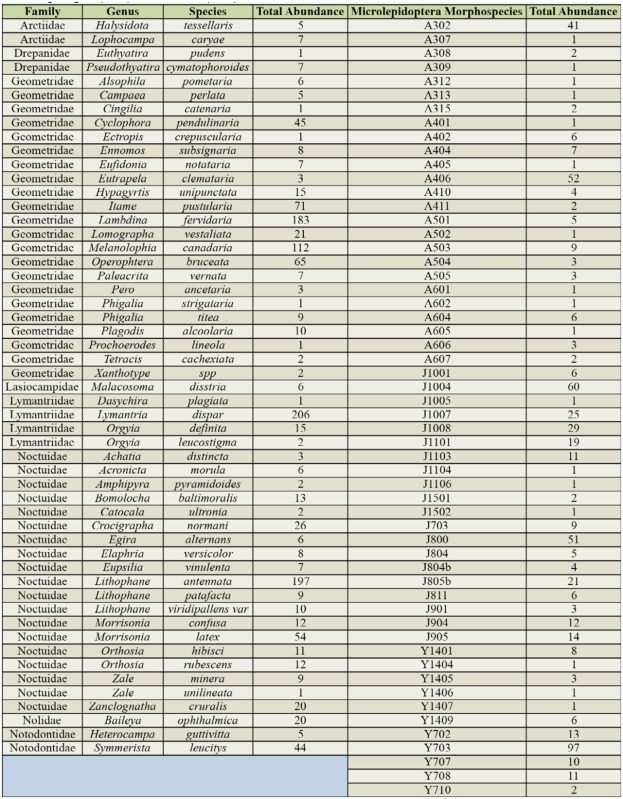
A record of the macrolepidoptera and microlepidoptera morphospecies surveyed. Microlepidoptera morphospecies were given unique alphanumeric designations, and subsequent individuals were verified with digital images. All macrolepidoptera were identified using Wagner (2005) and Handfield (1999).

**Appendix 4.**
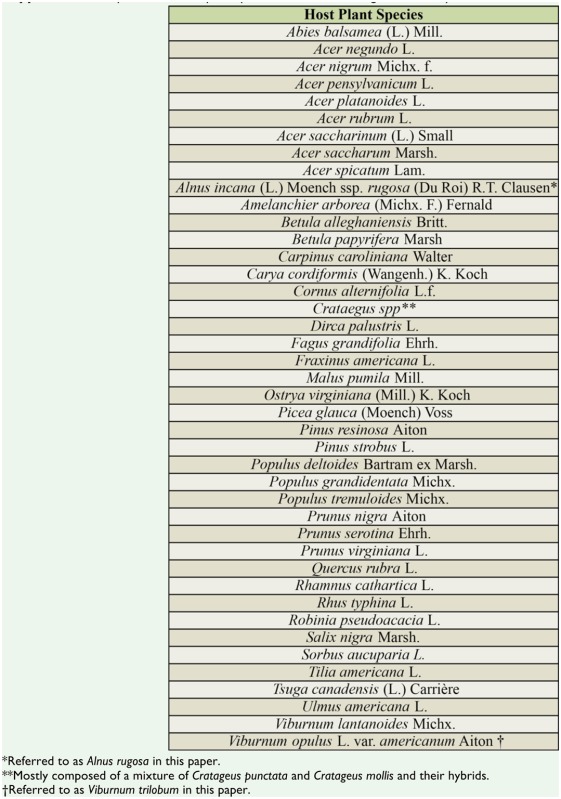
Complete list of host plant species documented in vegetation surveys.
